# Synthesis and Properties of a Lacquer Wax-Based Quarternary Ammonium Gemini Surfactant

**DOI:** 10.3390/molecules19033596

**Published:** 2014-03-24

**Authors:** Hongxia Chen, Chengzhang Wang, Jianzhong Ye, Hao Zhou, Li Lu, Zhibing Yang

**Affiliations:** 1 Institute of Chemical Industry of Forest Products, CAF, National Engineering Lab for Biomass Chemical Utilization; Key and Open Laboratory of Forest Chemical Engineering, SFA, Jiangsu Province, Nanjing 210042, China; 2 Institute of New Technology of Forestry, CAF, Beijing 100091, China; 3 Hubei Academy of Forestry, Wuhan, 430075, China

**Keywords:** *Toxicodendron vernicifluum*, lacquer tree, lacquer wax, fatty acids, surfactant, surface property

## Abstract

Lacquer wax is an important fatty resource obtained from the mesocarp of the berries of *Toxicodendron vernicifluum*. In order to expand the applications of lacquer wax, we hydrolyzed it after establishing the best conditions for the acid-catalyzed hydrolysis using a Box-Behnken design. Then we synthesized a quarternary ammonium gemini surfactant by a three-step reaction. The surface properties of an aqueous solution of the final product were investigated. The optimum conditions were 9% catalyst, 100 °C of reaction temperature and 14 h of reaction time, while the maximum free fatty acids (FFA)% was 99.67%. From the gas chromatography, the main fatty acids of the lacquer wax were palmitic, oleic and octadecanoic acid. The lacquer wax gemini surfactant was synthesized, and its structure was confirmed by IR and NMR. The experiments showed that the critical micelle concentration (CMC) is 5 × 10^−4^ mol·L^−1^, the surface tension is 33.6 mN·m^−1^. When the content of surfactant was 0.1%, the separation time of 5 mL water was 10 min.

## 1. Introduction

*Toxicodendron vernicifluum* is a deciduous forest tree belonging to the family *Anacardiaceae* [1]. Because of the raw lacquer, the trees have important economic and cultural value. The lacquer berries of *Toxicodendron vernicifluum* produce a lacquer wax and oil, which come from the testa and kernel of the berries. The lacquer wax is flexible, and can be used in lubricating agents, cosmetics and detergents. According to some Japanese research [2,3,4], the lacquer wax from the Japan contained hexadecanedioic acid, eicosanebioic acid and docosanedioic acid, which increased the elasticity and flexibility of the lacquer wax. In Japan, the lacquer wax was applied in high-end cosmetics, which had fine moisture retention. In China, some 150 million ton of lacquer berries are produced per year, so the output of lacquer wax is ample. In the provinces of Jiangxi, Hunan and Hubei, there are many *Toxicodendron vernicifluum* trees planted. In recent years, there have been many papers studying the lacquer berries. Dong [5] studied the thermal reflux processing of the berries by single factor and orthogonal experiments, analyzed the fatty acid composition of *Toxicodendron vernicifluum* berries from Shanxi and Jiangxi, and then researched the decoloration by physical adsorption [6]. After the decoloration, the whiteness of lacquer wax can reach 85.25. Zhang [7] used the lacquer lipids and various monosaccharides to synthesis sugar esters, which showed excellent emulsifying properties. In general, however, the development and utilization of lacquer wax are still lacking.

Gemini surfactants are a new generation of surfactants comprising two monomeric surfactant molecules chemically bonded together through a spacer near their head groups. They possess two hydrophilic and two hydrophobic groups. Relative to traditional surfactants, gemini surfactants exhibit higher surface activities and lower critical micelle concentrations (CMCs) [8]. In this work, a three-factor, three-level Box-Behnken design with temperature (X1), time (X2) and catalyst amount (X3) as the independent variables were selected for a study to optimize the hydrolysis of lacquer berry wax. The main fatty acids produced were identified by the corresponding GC and IR chromatograms. In order to expand the lacquer wax application, we then synthesized a quarternary ammonium gemini surfactant in a three-step reaction. The structure of the final product was confirmed by IR, ^1^H-NMR and ^13^C-NMR. The property of aqueous solutions of the final product was investigated. Through this study, we explore the high-value utilization of lacquer wax, improve the overall efficiency and extend the industrial chain of use of *Toxicodendron vernicifluum*, broadening the range of raw materials available as green surfactant sources.

## 2. Results and Discussion

### 2.1. Lacquer Wax Hydrolysis: Effect of Process Parameters and Statistical Analysis

The Box-Behnken design (BBD) [9] is a new analytical method for the optimization of processes. It presents the factors and responses involved during the optimization of analytical systems. It is a class of rotatable or nearly rotatable second-order designs based on three-level incomplete factorial designs. In this work, BBD was used to predict the levels of the factors temperature (X_1_), time (X_2_) and catalyst amount (X_3_). Table 1 shows the input parameters and experimental design levels used. The experimental design consists of a set of points lying at the midpoint of each edge and the replicated center points of the multidimensional cube. BBD was applied to the experimental data using the statistical software Design-expert version 7.1.3.

**Table 1:** Independent variables and their levels for Box-Behnken design of the hydrolysis reaction.

Independent Variables		Variable Levels		
		−1	0	1
Temperature (°C)	X_1_	90	100	110
Time (h)	X_2_	10	12	14
Catalyst amount (%)	X_3_	7	9	11

In order to study the combined effect of these factors, investigations were performed for different combinations of the physical parameters. Transformed values of all the batches along with their results are shown in Table 2. It also shows the observed and predicted values for all the batches. The free fatty acids (dependent variable) obtained at various levels of the three independent variables (X_1_, X_2_, and X_3_) were subjected to multiple regression to yield a second-order polynomial equation, as follows: 


FFA% = 98.96 + 6.70X_1_ + 2.57X_2_ + 0.35X_3_− 1.48X_1_X_2_− 2.25X_1_X_3_− 1.36X_2_X_3_− 8.30X_1_^2^− 1.95X_2_^2^− 0.66X_3_^2^ (1)

**Table 2:** Box-behnken design optimization of lacquer wax hydrolysis.

Run No.	Coded Independent Variable Levels			Observed FFA, %	Predicted
	Temperature (°C)	Time (h)	Catalyst Amount (%)		FFA, %
1	−1	−1	0	78.23	77.98
2	1	−1	0	94.19	94.33
3	−1	1	0	86.2	86.06
4	1	1	0	96.25	96.51
5	−1	0	−1	80.8	80.69
6	1	0	−1	99.1	98.59
7	−1	0	1	85.39	85.9
8	1	0	1	94.7	94.81
9	0	−1	−1	91.71	92.07
10	0	1	−1	99.67	99.92
11	0	−1	1	95.74	95.49
12	0	1	1	98.28	97.92
13	0	0	0	99.24	98.96
14	0	0	0	98.77	98.96
15	0	0	0	98.85	98.96
16	0	0	0	99.2	98.96
17	0	0	0	98.74	98.96

According to the regression model equations, the fitting coefficient of the three variables show values of 6.7 > 2.57 > 0.35, implying that the temperature and time are the main variables in the lacquer wax hydrolysis. After the BBD of the lacquer wax hydrolysis, the optimal conditions were a temperature of 100 °C, reaction time of 14 h, and catalyst amount of 9%, while the maximum free fatty acids was 99.67%. The statistical parameters obtained from the analysis of variance for the reduced models are given in Table 3. For the models, *p* < 0.001, implying that the models are significant, the models can predict the real experimental data. For the Pure error, *p* > 0.05, implying that the calculated values can be fit with the experimental values. The X_1_ and X_2_ variables have a significant effect on lacquer wax hydrolysis. For the X_1_X_2_ interaction, *p* < 0.01, implying that the temperature is closely related to time in the lacquer wax hydrolysis. For the X_1_X_3_ interaction, *p* < 0.01, implying that the temperature is closely related to the catalyst amount for the lacquer wax hydrolysis. For the X_2_X_3_ interaction *p* < 0.01, implying that the time is closely related to catalyst amount for the lacquer wax hydrolysis.

**Table 3:** Analysis of variance (ANOVA) of the response Y (FFA%) of the Box-Behnken design.

Sources of Variation	Sum of Squares	D*f*	Mean Square	*F*	*P*
Model	769.64	9	85.52	449.91	<0.001
X_1_	359.39	1	359.39	1890.77	<0.001
X_2_	52.69	1	52.69	277.18	<0.001
X_3_	1	1	1	5.27	0.0554
X_1_X_2_	8.73	1	8.73	45.94	0.003
X_1_X_3_	20.21	1	20.21	106.3	<0.001
X_2_X_3_	7.34	1	7.34	38.64	0.004
X_1_^2^	289.89	1	289.89	1525.13	<0.001
X_2_^2^	15.93	1	15.93	83.8	<0.001
X_3_^2^	1.86	1	1.86	9.8	0.0166
Residual	1.33	7	0.19		
Lack of fit	1.1	3	0.37	6.29	0.0538
Pure error	0.23	4	0.058		
total	770.97	16			

### 2.2. Gas Chromatography-Mass Spectrometery (GC-MS) Method Analysis of Fatty Acids Composition

The fatty acid methyl ester composition of the sample was identified by comparison with the Rtlpest3.L and Nist05.L mass spectral databases. Experimental results of the percentage of the composition of FAs are given in Table 4.

**Table 4:** Fatty acid composition after lacquer wax hydrolysis.

Fatty Acids	FA,%
Decanoic acid C10:0	0.107%
Pentadecanoic acid C15:0	0.09%
9-Hexadecenoic acid C16:1	0.093%
Hexadecanoic acid C16:0	71.722%
Heptadecanoic acid C17:0	0.088%
linoleic acid C18:2	0.104%
Oleic acid C18:1	11.505%
1,18-Octadecanedicarboxylic acid C20d+	0.229%
Octadecanoic acid C18:0	7.719%
Eicosanoic acid C20:0	0.037%
Hexadecanedioic acid C16d+	0.192%
11-Eicosenoic acid C20:1	1.35%
Arachidic acid C20:0	3.036%
Docosanoic acid C20	0.404%
Total saturated fatty acids (TSFA)	83.624%
Total unsaturated fatty acids (TUSFA)	13.052%
Total dibasic acids	0.421%

The main chemical components of lacquer wax are palmitic acid, oleic acid and octadecanoic acid, and the total fatty acids is 96.676%. The total amount of dibasic acids in the lacquer wax is 0.421%.

### 2.3. Synthesis of the Gemini Surfactant

**Scheme 1:**
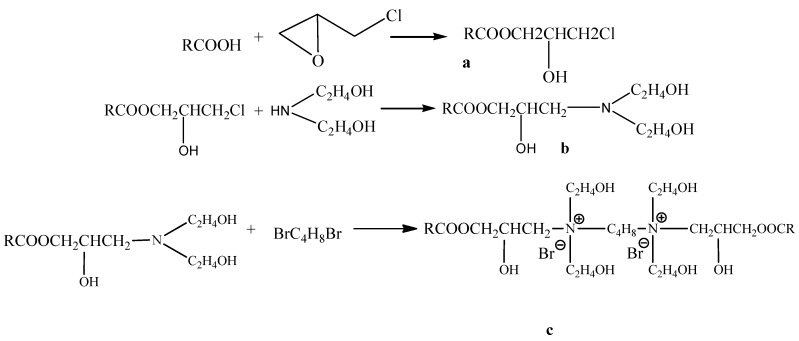
Synthetic route for the preparation of surfactant (R = fatty acids of lacquer wax).

The synthetic approach to target compounds c is outlined in Scheme 1. The compound contain the longer alkyl and quarternary ammonium groups, obtained in high yield. We monitored the course of reactions by IR spectroscopy. The structures of the products were confirmed NMR and IR analyses.

### 2.4. Surface Properties Analysis

**Figure 1:**
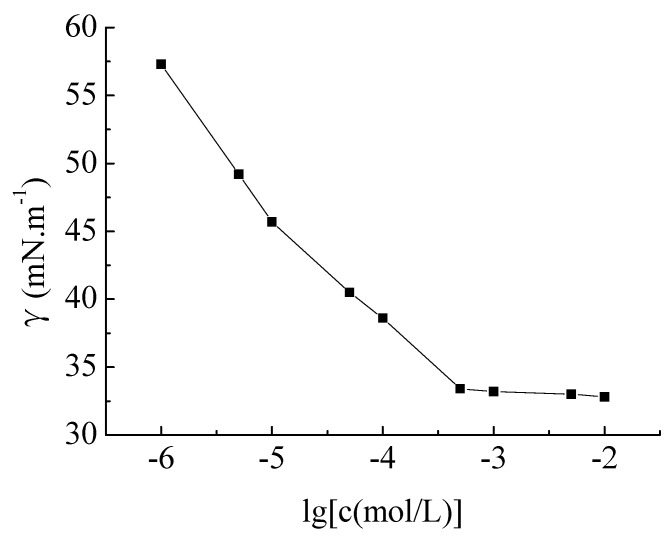
Surface tension (γ) (mN·m^−^^1^) *versus* logarithm of aqueous molar concentration (lg[c (mol/L)] of lacquer wax gemini surfactants at 25 °C.

The surface tension (γ) for aqueous solutions of the surfactant **c** at 25 °C *versus* log molar concentration (lgc) is shown in Figure 1. From the figure, the surface tension decreases as the concentration increases. At the break point, the surface tension is minimum, the product **c** forms micelles in the water. According to the curve, the CMC of the product **c** is 5 × 10^−4^ mol·L^−1^, and the surface tension is 33.6 mN·m^−1^. Compared with traditional surfactants [e.g., cetyltrimethyl ammonium bromide (CTAB)], it exhibits excellent surface activities. According to [10], the CMC of gemini surfactants decreases as the length of the alkyl group increases. The results thus match the reported results on the influence of alkyl groups for almost all conventional surfactants. When the length of the alkyl group increased, the hydrophobic performance was enhanced, promoting the close molecular arrangement at the air/water interface, and increasing the surfactant’s ability to form micelles. The main fatty acids of lacquer wax are palmitic acid, stearic acid, oleic acid and arachidic acid, *etc.* They all have long alkyl groups, and because the hydrophobic chains are closely packed, this effectively reduces the CMC. The structure of product **c** also contains hydroxyl groups, which form hydrogen bonds in aqueous solution, which can be closely connected to the the ionic head base, improving the surface activity.

**Figure 2:**
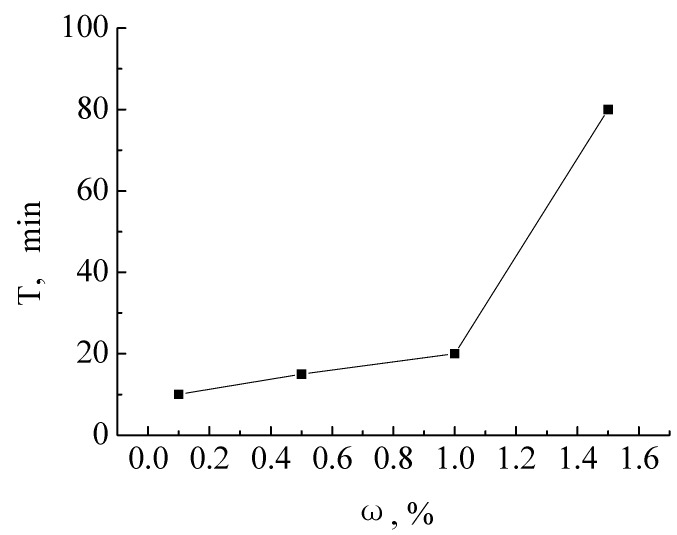
Emulsifying properities of lacquer wax gemini surfactants.

When the time for an emulsion to separate the same volume of water is longer, the emulsifying properties of the surfactant are stronger. Figure 2 shows the emulsifying properities curve of the different mass fractions of the product **c**. From the figure, the emulsifying properities increased as the mass fraction increased from 0.1% to 1.5%. This illustrated that product **c** could adsorb in the interface, forming a tenacious interfacial film, in order to delay the collisions of the dispersed beads to coagulate and condense. At the mass fraction of 0.1%, the time of separation of 5 mL of water is 10 min. 

The stability of the emulsions resulted from a combination of electrical repulsion and steric stabilization [11], the emulsifying ability of the surfactants was sensitive to the length of their alkyl chains. A decrease in the alkyl chain length resulted in an increase in the emulsifying ability. The fatty acids of the lacquer wax are complicated and large, which led to the poor emulsifying properities of the product **c**.

Foaming ability of the surfactant measures the foaming ability and stability of the resulting foam. The foaming ability of the product **c** is 50 mm, and after resting for 5 min, the foam height decreased to 20 mm. Therefore, the foaming ability and the foam stability of the product **c** are poor. It may be due to the hexadecanedioic acid and octadecanedicarboxylic acid of the lacquer wax, which made the reaction product chains longer, thus decreasing the adsorption ability at the gas-liquid interface, so it can’t effectively maintain the foam stability.

## 3. Experimental

### 3.1. Materials

Lacquer wax was obtained from HuBei Province. Sulfuric acid, sodium dodecyl sulfate (SDS), potassium hydroxide, epoxychloropropane, diethanolamine, sodium bicarbonate, tetrabutylammonium bromide, ethanol, ethyl acetate, 1,4-dibromobutane were obtained from Sinopharm Chemical Reagent Co. Ltd. (Nanjing, China).

### 3.2. Preparation of Lacquer Wax Hydrolyzate

Lacquer wax (50 g) was mixed in the reactor with distilled water (50 mL), then H_2_SO_4_ (50%) was added as catalyst and 1% sodium dodecyl sulfate (SDS) as emulsifier. The reactions were carried out in a 500 mL temperature-controlled reactor at different reaction temperatures and for different times. After the hydrolysis, the reaction product was washed with distilled water to neutral pH. The resulting lower layer was removed using a separating funnel and discarded. The FFA-containing upper layer was dried with anhydrous magnesium sulfate, and solvent was evaporated on a vacuum rotary evaporator at 45 °C. The free fatty acids of the lacquer wax were thus obtained and then the FFA% was calculated.

### 3.3. Synthesis of Quarternary Ammonium Gemini Surfactant

Step 1: Synthesis of products **a**: 

Lacquer wax fatty acids (10 g), tetrabutylammonium bromide (0.5 g) and epoxychloropropane (10 mL) were dissolved in ethanol (100 mL). The reaction mixture was heated at 90 °C for 10 h. After the reaction, the solvent was evaporated, and the residue was then washed several times and dried, to give a milky white solid, the yield was 85%. This is the first reaction in Scheme 1.

Step 2: Synthesis of products **b**: 

The product **a**, diethanolamine (10 mL) and sodium bicarbonate (1.5 g) were dissolved in ethanol (100 mL). Then the mixture was reacted at 90 °C for 6 h. After the reaction, the solvent was evaporated and the residue dissolved in ethyl acetate and filtered. The filtrate was evaporated to give a pale yellow extract (90% yield). The synthesis of **b** is shown in the second reaction of Scheme 1.

Step 3. Synthesis of products **c**:

The product **b** and 1,4-dibromobutane (20 mL) were dissolved in ethanol (100 mL) and then reacted at 100 °C for 48 h. After the reaction, the solvent was evaporated to give a brown viscous liquid. Then re-dissolved in the ethyl acetate, obtained the insoluble fraction, and drying with anhydrous magnesium sulfate, a faint yellow viscous liquid was obtained (75% yield). The synthesis of **c** is shown in the third reaction of Scheme 1.

### 3.4. Determination of the FFA%

The FFA% of the lacquer wax hydrolysis was determined according to Equations (2) and (3). The saponification value and acid value were measured according to standard methods [12,13]:

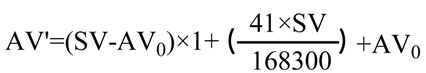
(2)

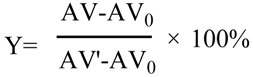
(3)
where:

AV' ‒ The theoretical acid value of the wax, as KOH, mg/g;
AV ‒ The acid value of the hydrolyzate of the wax phase, as KOH, mg/g;
AV_0_‒ The acid value of the raw wax with KOH, mg/g;
SV ‒ The saponification value of the raw wax with KOH, mg/g;
Y – Percentage free fatty acids of the hydrolyzate of the wax,%.

### 3.5. Gas Chromatography-Mass Spectrometry (GC-MS) Fatty Acids Analysis

The fatty acids constituents were analyzed by GC-MS. This was performed on a Aglient Technologies 6890/5973 instrument equipped with a flame ionization detector and capillary column (HP-5 MS 30 m × 0.25 mm × 0.25 μm). Injection port temperature 250 °C, Column initial temperature 110 °C, Insulation 10 min, 10 °C/min rose to 220 °C, Insulation 18 min, The injection volume 1 μL. The ion source was operated in the electron impact mode with 70 eV electron energy, the temperature was at 230 °C, the transfer line was at 280 °C, and the photomultiplier was set to 2,200 V. The spectra were collected at 3 scans/s over the mass rang (*m/z*) 29–450 u.

### 3.6. Structural Analytical Methods

FTIR of the products were recorded on a Perkin Elmer Spectrum GX spectrophotometer in the range 400–4,000 cm^−1^. FTIR was used to determine the functional groups present in the FAs, and products **a**, **b** and **c**. Structures of the products **c** were confirmed by the ^1^H-NMR and ^13^C-NMR, recorded of the sulutions in CDCl_3_ using a Bruker DRX300 spectrometer.

### 3.7. Spectral Data

The IR spectra of all the lacquer wax hydrolyzates showed absorption bands at 2,915, 2,847 cm^−^^1^ (C-H stretching), 1,700 cm^−^^1^ (C=O stretching), 1,462 cm^−^^1^ (C-H bending), 1,227 cm^−^^1^ (C-O stretching) and 722 cm^−^^1^ (C-H rocking).

The IR spectra of the intermediates **a** (Scheme 1) showed absorption bands at 3,490 cm^−^^1^ (-OH stretching), 2,913, 2,849 cm^−^^1^ (C-H stretching), 1,735 cm^−^^1^ (C=O stretching), 1,469 cm^−^^1^ (C-H bending), 1,179 (C-O stretching), 1,114 cm^−^^1^ (C-N stretching) and 716 cm^−^^1^ (C-H rocking).

The IR spectra of all intermediates **b** (Scheme 1) showed absorption bands at 3,361 cm^−^^1^ (-OH stretching), 2,919, 2,850 cm^−^^1^ (C-H stretching), 1,738 cm^−^^1^ (C=O stretching), 1,621 cm^−^^1^ (C=C stretching), 1,465 cm^−^^1^ (C-H bending), 1,375 cm^−^^1^ (O-H bending), 1,176 cm^−^^1^ (C-O stretching), 1,045 cm^−^^1^ (C-N stretching), 716 cm^−^^1^ (C-H rocking).

IR spectra of the lacquer wax gemini surfactants **c** showed absorption bands at 3,317 cm^−^^1^ (-OH stretching), 2,955 cm^−^^1^ (C-H stretching), 1,737 cm^−^^1^ (C-H stretching), 1,446 cm^−^^1^ (C-H bending), 1,235 cm^−^^1^ (C-O stretching), 1,056 cm^−^^1^ (C-N stretching), 931 cm^−^^1^ (C-N stretching), 784 cm^−^^1^ (C-H rocking).

^1^H-NMR of the lacquer wax gemini surfactants **c** (CDCl_3_): δ 4.44 (4H, s, ‒C(=O)O‒CH_2_‒), δ 3.99 (2H, m, >CH‒), δ 2.08 (2H, d, >C(OH)‒), δ 1.16 (4H, m, >C‒CH_2_‒N), δ 3.69 (8H, t, R_3_N‒CH_2_‒), δ 3.03 (8H, m, N‒C‒CH_2_‒O), δ 2.55 (4H, s, ‒C‒C‒OH), δ 3.15 (4H, t, N‒CH_2_‒C‒), δ 1.69 (4H, m, N‒C‒CH_2_‒C‒C).

^13^C-NMR of the lacquer wax gemini surfactants **c**: δ 72.45 (‒O‒CH_2_‒), δ 65.8 (‒CH(OH)‒), δ 64.78 (>C‒*C*H_2_‒N), δ 63.63 (R_3_N‒CH_2_‒CH_2_‒OH), δ 63.28 (R_3_N‒CH_2_‒CH_2_‒OH), δ 38.66 (R_3_N‒CH_2_‒CH_2_‒CH_2_‒CH_2_‒NR_3_), δ 39.5 (R_3_N‒CH_2_‒CH_2_‒CH_2_‒CH_2_‒NR_3_).

### 3.8. Surface Properties Analytical Methods

The surface tensions of aqueous solutions of the surfactant product **c** at different concentrations were measured at 25 ± 0.1 °C by the ring method [14], repeated three times. The surface tension (mN·m^−^^1^) is plotted *versus* the logarithm of the aqueous molar concentration (log C mol/L) curve (δ-C). The break point is the critical micelle concentration (CMC).

The emulsification properties were investigated by mixing in a graduated cylinder at room temperature [15]. An aqueous surfactant solution (20 mL) containing different concentrations of the product **c** and hexane (20 mL) were poured into a 100 mL cylinder. Then it was inverted up and down sixty times, rest and timing, and the time needed for 5 mL of water to separate was recorded. This was repeated three times.

The foaming properties were investigated by mixing in a graduated cylinder at room temperature [16]. An aqueous surfactant solution (20 mL) containing 0.5% of product **c** was poured into a 100 mL cylinder, then water (10 mL) was added, inserting a plug. Then it was inverted up and down 25 times, the foam height was recorded, and then allowed to stand for 5 min, observing the foam performance.

## 4. Conclusions

Lacquer wax is an important fatty resource obtained from the mesocarp of the berries of *Toxicodendron vernicifluum*. There are many lacquer wax resources in the China, but they still lack utilization, so in order to expand the applications of lacquer wax. First we hydrolysed the lacquer wax, obtaining a fatty acid mixture, and then we successfully synthesized a quarternary ammonium gemini surfactant by a three-step reaction. We determined the optimum hydrolysis conditions: catalyst amount 9%, 100 °C of reaction temperature and 14 h of reaction time, and a maximum FFA% of 99.67% was obtained. By the gas chromatography, the main fatty acids of the lacquer wax were palmitic, oleic and octadecanoic acid. According to the surface properties analysis, the critical micelle concentration (CMC) of the resulting lacquer wax gemini surfactant is 5 × 10^−4^ mol·L^−1^, and the surface tension is 33.6 mN·m^−1^. Through simple steps and reactions, we have obtained the lacquer wax based surfactant, which has good surface activity.

## References

[1] Zheng M., Min T.L. (1980). Flora of China-Anacardiaceae.

[2] Shichiro S. (1946). Dibasic acid contajned in the wax of Rhus tricocarpa and R. succedanea. Soc. Chem. Ind. Jpn..

[3] Sitiro S. (1940). The dicarboxylic acids of Japan wax. Soc. Chem. Ind. Jpn..

[4] Kiyoshi K., Tsuneturo K. (1952). The composition and crystal of the dibasic acids of Japan wax. Chem. Soc. Jpn. Ind. Chem. Sect..

[5] Dong Y.H., Wang C.Z., Ye J.Z. (2010). Extraction technology and its chemical composition of the lacquer wax. J. Beijing For. Uni..

[6] Dong Y.H., Wang C.Z., Chen H.X. (2011). Adsorption decolourization process research of lacquer wax. Chem. Ind. For. Prod..

[7] Zhang F.L., Zhou Z.L. (2012). Lacquer Wax Sugar Ester and Its Preparation Method. Patent.

[8] Tomokazu Y., Kunio E. (2004). Synthesis and surface properties of anionic gemini surfactants with amide groups. J. Colloid Interf. Sci..

[9] Ferreiraa S.L.C., Brunsb R.E., Ferreiraa H.S. (2007). Box-Behnken design: An alternative for the optimization of analytical methods. Anal. Chim. Acta.

[10] El Achouri I.M., Bensouda Y., Gouttaya H.M., Nciri B., Perez L., Infante M.R. (2001). Gemini surfactants of the type 1,2-ethanediyl bis-(dimethylalkylannonium bromide). Tenside Surfactants Deterg..

[11] Lin L.H., Chou Y.S. (2010). Surface activity and emulsification properties of hydrophobically modified dextrins. Colloids Surf. A Physicochem. Eng. Aspects.

[12] Zhang S.H. (2008). Animal and Vegetable Fats and Oils-Determination of Saponification Value.

[13] Xue Y.L. (1996). Animal and Vegetable Fats and Oils-Determination of Acid Value and Acidity.

[14] Zhuang Y.B., Cao D. (2007). Surface Active Agents-Determination of the Critical Micellization Concentration.

[15] Mao P.K. (1988). Analysis of the Synthetic Detergent Industry.

[16] Xu M., Zhang S.Q. (1994). Surface Active Agents-Measurement of Foaming Power—Modified Ross-Miles Method.

